# The microbiological diagnosis of pediatric tuberculosis: focus on the Ultra test.

**DOI:** 10.36416/1806-3756/e20250241

**Published:** 2026-03-05

**Authors:** Claudia Stella Pereira Battaglia, Rafaela Baroni Aurilio, Michley Alexandrino Pinheiro, Ana Alice Amaral Ibiapina Parente, Ronir Raggio Luiz, Clemax Couto Sant´Anna, Maria de Fatima B. Pombo Sant´Anna

**Affiliations:** 1. Instituto de Puericultura e Pediatria Martagão Gesteira, Universidade Federal do Rio de Janeiro, Rio de Janeiro (RJ), Brasil.; 2. Faculdade de Medicina, Universidade Federal do Rio de Janeiro, Rio de Janeiro (RJ), Brasil.; 3. Instituto de Estudos em Saúde Coletiva, Universidade Federal do Rio de Janeiro, Rio de Janeiro (RJ), Brasil.

**Keywords:** tuberculosis, real-time PCR, children, adolescents

## Abstract

**Introduction:**

GeneXpert^®^ MTB/RIF Ultra (Ultra) is a method for detecting the Mycobacterium tuberculosis complex (Mtb) with greater sensitivity compared to GeneXpert^®^ MTB/RIF (Xpert). In Brazil, for children and for extrapulmonary tuberculosis (EPTB), “traces detected” results are considered positive for Mtb. Here, we describe the use of Ultra at a reference center for pediatric tuberculosis (TB) in Rio de Janeiro, Brazil.

**Methods:**

A cross-sectional study was conducted with children (0-9 years) and adolescents (10-18 years) with presumed EPTB or pulmonary TB (PTB) whose specimens were tested using Ultra. Data were analyzed using descriptive statistics, and Fisher’s exact test (significance level p<0.05) was applied when appropriate. The study was approved by the Ethics Committee of IPPMG-UFRJ (CAAE: 02173518.2.0000.5264).

**Results:**

Ninety-three patients with presumed TB were included: 44% with PTB and 56% with EPTB. The final diagnoses revealed 63.4% PTB, 40.4% EPTB, and 49.5% other diagnoses. Among the PTB cases, 9/26 (34%) had positive results exclusively by Ultra. As for the EPTB cases, 11/21 (52%) were positive only by Ultra. For PTB, sensitivity was 50% and specificity was 100% (compared to culture). For EPTB, sensitivity was 85.7% and specificity was 100%.

**Conclusion:**

Ultra contributed to the diagnosis of both PTB and EPTB, especially in cases with negative culture results. The test demonstrated higher sensitivity in EPTB than in PTB.

## INTRODUCTION

In order to enhance the laboratory diagnosis of tuberculosis (TB), the World Health Organization (WHO) recommended in 2017 that the GeneXpert^®^ MTB/RIF (Xpert) rapid molecular assay be replaced by the GeneXpert^®^ MTB/RIF Ultra (Ultra) assay.^1^ ^-^ ^3^ This new protocol was implemented in Brazil in October 2019. Ultra results categorize the bacillary load as “detected,” “not detected,” or “traces detected” (with an indeterminate rifampicin resistance result). According to the WHO, in people living with HIV (PLHIV), children under ten years of age, and in cases of extrapulmonary TB (EPTB), “traces detected” results are considered positive.^1^ ^,^ ^2^ ^,^ ^4^

A systematic review and meta-analysis on pediatric TB reported that the Xpert assay had a sensitivity and specificity of 64.6% and 99.0%, respectively, whereas the Ultra assay showed 72.8% sensitivity and 97.5% specificity.^5^ Nevertheless, most studies using the Ultra assay have focused on adults, whose lesions typically contain a higher bacillary load than those observed in children. The aim of the present study was to evaluate the microbiological diagnosis of pediatric pulmonary TB (PTB) and EPTB, focusing on the performance of the Ultra assay in a pediatric university hospital in Rio de Janeiro (RJ), Brazil.

## METHODS

This observational, cross-sectional, descriptive study included prospective data collected from children (0-9 years) and adolescents (10-19 years) with presumed PTB and EPTB who were tested with the Xpert MTB/RIF Ultra assay. The study was conducted between January 2020 and December 2022 at the Martagão Gesteira Institute of Pediatrics and Childcare of the Federal University of Rio de Janeiro (UFRJ), a reference pediatric TB center in Rio de Janeiro, Brazil. The analyzed variables included age, sex, nutritional status (measured as weight-for-age percentile based on the Centers for Disease Control and Prevention (CDC) growth charts), the Brazilian Ministry of Health (MoH-Brazil) TB score (>40 points = very likely TB; 30-35 points = possible TB; <25 points = unlikely TB), acid-fast bacilli (AFB) detection in Ziehl-Neelsen smears, *Mycobacterium tuberculosis* culture, and human immunodeficiency virus (HIV) antibody testing.^6^ ^-^ ^8^

The final diagnosis of PTB was established based on clinical and radiological findings and clinical response after two months of treatment initiation. Children and PLHIV with a “traces detected” result were considered positive.^7^ ^,^ ^8^ Patients were classified into three groups: Group 1, confirmed TB (microbiological confirmation); Group 2, unconfirmed TB (clinical diagnosis without microbiological confirmation); and Group 3, non-TB.

Samples were collected and processed for the Ultra assay, AFB testing, culture using the Mycobacteria Growth Indicator Tube (MGIT) system, and antibiotic susceptibility testing (AST) if the MGIT culture was positive, at the Mycobacteriology Laboratory of UFRJ. 

The sensitivity, specificity, positive predictive value (PPV), and negative predictive value (NPV) of the Ultra assay were calculated separately for the PTB and EPTB groups. All data were coded and entered into a database using Excel 12.0 (Office 2007) and analyzed with SPSS software, version 20.0 (IBM Corp., Armonk, NY, USA). Categorical variables were assessed using descriptive statistics and expressed as frequencies and proportions. Ultra assay results were analyzed as categorical variables (“detected,” “not detected,” and “traces detected”). Fisher’s exact test was used to compare groups, and p-values < 0.05 were considered statistically significant. McNemar’s statistical test was used to compare Ultra assay results with presumptive diagnoses.

The study was approved by the Research Ethics Committee of the Martagão Gesteira Institute of Pediatrics and Childcare, UFRJ (CAAE No. 02173518.2.0000.5264).

## RESULTS

A total of 93 patients were included: 41 (44%) with presumed PTB and 52 (56%) with presumed EPTB; none of the cases were excluded from the study. Among these patients, 26/41 (63.4%) had a final diagnosis of PTB, 21/52 (40.4%) had EPTB, and 46/93 (49.5%) received other diagnoses. The characteristics of the study population are shown in Table 1. In Groups 1 and 2, considering “traces detected” results as positive in children under 10 years of age and/or PLHIV, the sensitivity and specificity of the Ultra assay-when the result was “detected” or “traces detected”-were 50% (13/26) and 100% (15/15), respectively. In Group 3, values ​​of 85.7% (18/21) and 100% (31/31) were observed using the same criteria for defining positive results. 

**Table 1:** Characteristics of the population according to final diagnosis of TB.

	Total		Final diagnosis			
			TB (Groups 1 and 2)		Non-TB (Group 3)	
	(n = 93; 100%)		(n = 47; 50.5%)		(n = 46; 49.5%)	
	n	%	n	%	n	%
Presumed diagnosis						
PTB	41	100.0	26	63.4	15	36.6
EPTB	52	100.0	21	40.4	31	59.6
HIV						
Positive	3	100.0	2	66.7	1	33.3
Negative*	90	100.0	45	50.0	45	50.0
Sex						
Male	51	100.0	24	47.1	27	52.9
Female	42	100.0	23	54.8	19	45.2
Age (years)						
0 - 9	56	100.0	25	44.6	31	55.4
10 - 17	37	100.0	22	59.5	15	40.5
Nutritional status (weight/age)						
< P_3_	22	100.0	13	59.1	9	40.9
P_3_ - P_10_	8	100.0	3	37.5	5	62.5
> P_10_	63	100.0	31	49.2	32	50.8

PTB, pulmonary tuberculosis; EPTB, extrapulmonary tuberculosis. *Included the 10 unrecorded cases of HIV.

The PPV and NPV of the Ultra assay in presumed PTB cases were 100% (13/13) and 53.6% (15/28), respectively, based on a prevalence of 63.4%. For presumed EPTB, the PPV and NPV were 100% (18/18) and 91.2% (31/34), respectively, with a prevalence of 40.4%. The Ultra assay yielded negative results in 13/28 (46%) patients with presumed PTB and in 3/34 (9%) patients with presumed EPTB (Table 2). When analyzing the quantitative results of the Ultra assay, 7/7 (100%) patients with a “detected” result, as well as 11/12 (92%) with a “traces detected” result, were diagnosed with PTB. The only patient with a “traces detected” result who was not diagnosed with PTB was an adolescent with significant oncohematological comorbidities whose respiratory symptoms improved with the use of common antibiotics within two weeks; therefore, this case was considered a false positive. 

**Table 2:** Children and adolescents with presumed TB (n = 93), according to Ultra assay results and final diagnosis.

Presumed diagnosis	Ultra result	Total		Final diagnosis				Predictive values		p-value (McNemar)
				TB		Non-TB				
		n	%	n	%	n	%	Positive	Negative	
PTB	Positive*	13	31.7	13	50.0	0	0	100%	53.6%	< 0.001
	Negative	28	68.3	13	50.0	15	100			
	Total	41	100.0	26	63.4	15	36.6			
EPTB	Positive*	18	34.6	18	85.7	0	0	100%	91.2%	0.250
	Negative	34	65.4	3	14.3	31	100			
	Total	52	100.0	21	40.4	31	59.6			

TB, tuberculosis; PTBP, pulmonary tuberculosis; EPTB, extrapulmonary tuberculosis. *Positive = Ultra assay ‘detected’ and ‘traces detected’ in children (< 10 years of age) and people living with HIV (PLHIV).

It was observed that 28/41 (68%) patients with presumed PTB had negative Ultra results. Of these, 13/28 (46%) were diagnosed with PTB, with 12/13 (92%) in Group 2 and 1/13 (8%) in Group 1. The diagnosis of patients in Group 2 was based on the MoH-Brazil score, as they tested negative in microbiological assays. Among the 13 patients with PTB and negative Ultra results, 9/11 (82%) were classified as “very likely TB” or “possible TB,” 2/11 (18%) as “unlikely TB,” and 2/13 (15%) had incomplete data for scoring. 

When evaluating data from patients with presumed EPTB, all individuals with a “detected” or “traces detected” result were confirmed to have EPTB, representing 83% of the positive final diagnoses obtained using this method. Among the 34 patients with negative Ultra results, only 3/34 (46%) were ultimately diagnosed with EPTB.

When using culture as the reference standard in PTB cases, the PPV and NPV were 66.7% and 100%, respectively. Ultra assay positivity was 30.8%, with a specificity of 76.5% and sensitivity of 80%. For EPTB, the PPV and NPV were 87.7% and 100%, respectively, as shown in Table 3.

**Table 3:** Positive and negative predictive values (PPV and NPV) in children and adolescents with presumed TB (PTB and EPTB), according to the final diagnosis and using culture as the reference standard.

Positive and negative predictive values	Ultra	Total		Culture			
				Positive		Negative	
		(n = 90*)		(n = 12)		(n = 78)	
		n	%	n	%	n	%
PPV and NPV	Detected	6	100.0	4	66.7	2	33.3
	Not detected	21	100.0	0	0.0	21	100.0
	Traces detected	12	100.0	1	8.3	11	91.7
PPV and NPV	Detected	8	100.0	7	87.5	1	12.5
	Not detected	33	100.0	0	0	33	100.0
	Traces detected	10	100.0	0	0	10	100.0

PTB, pulmonary tuberculosis; EPTB, extrapulmonary tuberculosis. *The 3 cases with contaminated culture were excluded - 1 with EPTB and 2 with PTB.

Among the 93 samples analyzed with the Ultra assay, two distinct specimen groups were identified in PTB: 34/41 (83%) respiratory samples and 7/41 (17%) pleural samples. The predominant specimens in EPTB were 21/52 (40%) lymph node samples and 9/52 (17%) cerebrospinal fluid samples. In the respiratory samples, Ultra positivity was 35.3%, while in the pleural samples it was 14.3%. For EPTB, Ultra positivity reached 43% in lymph node samples and 11% in cerebrospinal fluid samples.

In order to provide a more accurate assessment of the Ultra assay’s diagnostic efficacy, the results were compared based on the presence or absence of other simultaneous positive diagnostic tests. Among the 26/41 (63%) patients diagnosed with PTB (Groups 1 and 2), 9/26 (34%) had positive results exclusively in the Ultra assay (negative AFB smears and culture). Similarly, among the 21/52 (40%) patients diagnosed with EPTB, 11/18 (61%) were positive only in the Ultra assay, also with negative AFB and culture results. 

## DISCUSSION

Our data indicate that the Ultra assay was a useful diagnostic tool, especially in patients for whom other methods, such as culture and AFB smears, were negative. The test showed higher sensitivity in EPTB than in PTB, while the rate of false-negatives was higher in PTB (46%) than in EPTB (9%). It was not possible to evaluate the occurrence of more severe clinical forms in PLHIV, as reported in the literature, because there were only three such patients in our sample.

The use of diagnostic tests that do not depend on culture to confirm pediatric TB has been formally recommended by the WHO in recent years, with the incorporation of nucleic acid amplification tests (NAAT), among other methods. Consequently, GeneXpert^®^ MTB/RIF and later GeneXpert^®^ MTB/RIF Ultra have become attractive tools for diagnosing pediatric TB, particularly in children, given the difficulty of isolating *M*. *tuberculosis* from clinical specimens.^5^

In our case series, 92% of patients with PTB and 83% of those with EPTB had a “detected” or “traces detected” result. The MoH-Brazil follows the WHO recommendation that, in children under 10 years of age, a “traces detected” result should be considered positive.^7^ In Uganda, a community-based TB screening study using sputum Ultra found that more than half of the individuals over 15 years of age with a “traces detected” result were confirmed to have TB within two years. These patients were either asymptomatic or had mild symptoms but displayed chest radiological abnormalities. The risk of developing TB remained high, even among those extensively investigated at baseline to rule out the disease. Thus, a “traces detected” result in adolescents and adults represents a significant risk for active TB.^9^ Another study, also from Uganda, assessing adolescents and adults with “traces detected” results in sputum during initial PTB investigations, demonstrated that 48% developed active TB within three months of testing.^10^

The WHO recommends that, in individuals over 10 years of age, a “traces detected” result should not be considered diagnostic of active disease unless the patient is HIV-positive. This highlights the need for a rigorous clinical evaluation before ruling out active TB. Ultra-negative results (whether “not detected” or “traces detected” in adolescents) do not exclude the disease. Moreover, adolescents are more likely to have a previous history of TB, which may contribute to an increased rate of false positives associated with “traces detected” results. In light of the Ugandan studies and a recent review,^9^ ^,^ ^11^ it may be worth considering extending the WHO recommendation to adolescents, both globally and in Brazil. 

We chose to include the MoH-Brazil score as one of the criteria for the final diagnosis in this study, as it has been validated in previous research demonstrating high sensitivity and specificity. In addition, the two-month follow-up period allowed greater confidence in confirming each participant’s final diagnosis.^6^ ^,^ ^7^ The low sensitivity of 50% observed in PTB in this study had already been reported by Battaglia et al. (2025) in another paper by our group. Accordingly, the authors emphasized the importance of using complementary diagnostic approaches for PTB rather than relying solely on molecular testing, which is consistent with the literature.^1^ ^,^ ^2^ ^,^ ^6^ On the other hand, the sensitivity of 86% (compared to culture) observed in EPTB in our study supports the value of testing multiple specimen types for accurate diagnosis in children and adolescents.^12^

Although it is challenging to define specific sensitivity values ​​for the various forms of EPTB, our results are consistent with the meta-analysis by Gong et al. (2023), which reported a sensitivity of 84% (95% CI: 76-90) for peripheral lymph node TB using lymph node biopsy samples. The authors evaluated the Xpert assay rather than Ultra, which is known to be more sensitive. Conversely, that same meta-analysis reported lower sensitivity values ​for TB of the nervous system (60%) and pleural TB (30%).^13^ In Bangladesh, a study involving children under 14 years of age showed that cerebrospinal fluid testing with Ultra detected 26 of 44 children with TB meningoencephalitis who had negative results on other tests, with 85% of these showing “traces detected” results.^14^

The present study has some limitations. It was conducted at a single reference center, without inclusion of primary healthcare facilities, and the relatively small sample size limits the generalizability of the findings, although some results reached statistical significance. Likewise, comparison between Ultra and Xpert was not possible, as Ultra replaced Xpert in Brazil in 2019. Nonetheless, this is one of the few Brazilian studies assessing the diagnostic performance of Ultra in pediatric TB. Furthermore, it reinforces that the MoH-Brazil score remains a valuable diagnostic tool for children and adolescents.
